# Prenatal breastfeeding knowledge, attitude and intention, and their associations with feeding practices during the first six months of life: a cohort study in Lebanon and Qatar

**DOI:** 10.1186/s13006-022-00456-x

**Published:** 2022-02-24

**Authors:** Farah Naja, Aya Chatila, Jennifer J. Ayoub, Nada Abbas, Amira Mahmoud, MINA collaborators, Mariam Ali Abdulmalik, Lara Nasreddine

**Affiliations:** 1 Clinical Nutrition and Dietetics Department, College of Health Sciences, University of Sharjah, Sharjah, UAE; 2 Research Institute for Medical and Health Sciences, University of Sharjah, Sharjah, UAE; 3 Faculty of Agriculture and Food Sciences, American University of Beirut, Beirut, Lebanon; 4 Nutrition and Food Sciences Department, Faculty of Agriculture and Food Sciences, American University of Beirut, Beirut, Lebanon; 5 Public Health Department, Ministry of Public Health, Doha, Qatar; 6 Primary Health Care Corporation, Doha, Qatar

**Keywords:** Breastfeeding, Feeding practices, Associations, Knowledge, Attitude, Exposure, Intention, Lebanon, Qatar

## Abstract

**Background:**

Prenatal knowledge, attitude, and intention related to breastfeeding are postulated as important modulators of feeding practices. Using data from the Mother and Infant Nutritional Assessment (MINA) study, a three year cohort conducted in Lebanon and Qatar, this study aimed to characterize breastfeeding practices during the first six months postnatally and examine their associations with prenatal breastfeeding knowledge, attitude, exposure, and intention.

**Methods:**

Pregnant women during their first trimester were recruited from primary healthcare centers in Beirut and Doha. Data collection was conducted in 2015 − 2018. Participants were followed-up until the child was twoyears old. Exposure, knowledge, attitude, and intentions regarding breastfeeding were assessed during the third trimester of pregnancy (*n* = 230), using validated questionnaires and scales. Breastfeeding practices were evaluated at four months (*n* = 185) and six months (*n* = 151) postpartum. Early initiation of breastfeeding was defined as putting the infant to the breast within one hour of birth, and exclusive breastfeeding (EBF) as feeding exclusively with breast milk.

**Results:**

Breastfeeding practices were as follows: ever breastfeeding: 95.8%; early initiation of breastfeeding: 72.8%; breastfeeding at four and six months: 70.3% and 62.3%; EBF at four and six months: 35.7% and 18.5%. Over 95% of participants had high breastfeeding exposure, and 68.8% had strong / very strong intentions to breastfeed. Only 25% had very good knowledge, and 9.2% reported positive/strong positive attitude towards breastfeeding. After adjustment, high exposure was associated with greater odds of breastfeeding initiation (OR 10.1: 95% CI 1.25, 80.65). Both positive attitude towards breastfeeding and strong intention to breastfeed were associated with EBF at four months (OR 2.51; 95% CI 1.02, 6.16 and OR 4.0; 95% CI 1.67, 9.6), breastfeeding at four months (OR 2.92: 95% CI 1.29, 6.62 and OR 5.00: 95% CI 2.25, 11.1), and breastfeeding at six months (OR 3.74: 95% CI 1.24, 11.32 and OR 8.29: 95% CI 2.9, 23.68).

**Conclusions:**

Findings of this study documented suboptimal knowledge and attitude towards breastfeeding and showed that prior exposure, a positive attitude, and a strong intention to breastfeed prenatally were significant predictors of breastfeeding practices postnatally. This highlights the need to develop specific interventions and policies aimed at improving breastfeeding attitudes and creating an enabling environment that supports women throughout their breastfeeding journey.

**Supplementary Information:**

The online version contains supplementary material available at 10.1186/s13006-022-00456-x.

## Background

Adequate nutrition during the first 1000 days of life [1], has been recognized as a window of opportunity for fostering optimal health and development, while also reducing the risk of non-communicable diseases (NCDs) later in life [2, 3]. The World Health Organization (WHO) recommends breastfeeding (BF) newborns within one hour after birth, exclusive breastfeeding (EBF) for the first six months of life, and continued BF until two years of age with appropriate complementary feeding initiated at six months [4]. EBF for the first six months of life was found to promote sensory, cognitive and socio-emotional development in infants, decrease the risk of respiratory and gastro-intestinal infections, improve growth, and reduce the risk of stunting [5–14]. Despite the evidence supporting the importance of BF for child health, early life feeding practices remain suboptimal at the global level [15, 16]. To improve infant and young child nutrition, the World Health Assembly endorsed, in 2012, the WHO global nutrition targets, which include increasing the rate of EBF in the first six months up to at least 50% in 2025 (Target 5) [17].

As infant feeding decisions appear to be made prenatally [18], pregnant women represent a key population of interest for characterizing the culturally prevalent norms, knowledge, and attitude towards BF, and for identifying misconceptions and negative perceptions that may lead to inadequate BF practices [19–21]. Studies have shown that the determinants of BF initiation, duration, and exclusivity are multifactorial and operate at multiple levels [10, 11, 22–29]. They include demographic and socioeconomic factors such as maternal age, education, parity, monthly income, and mother’s working status [23]; community support and structural factors; sociocultural beliefs and misconceptions prevalent in the community and among healthcare practitioners [24─26], as well as personal factors such as knowledge about the benefits of BF [27]. The theory of planned behavior has been extensively used to predict BF practices in various cultural settings [30]. According to this theory, BF intention is a direct precursor to BF behavior and practices. The intention to breastfeed is in turn influenced by maternal knowledge and attitude towards BF as well as the mother’s prior exposure to BF [31, 32]. Several cross-sectional studies conducted among pregnant women have established a link between BF exposure, knowledge, and attitude, with the intention to BF prenatally [20, 23, 29, 33]. However, few studies have investigated, longitudinally, the association between these maternal prenatal attributes and actual BF practices postnatally [22, 34]. This is important given that some studies have shown that, even among women who expressed their intention to breastfeed, few were able to achieve their intended BF or EBF duration [35]. Gaining a deeper understanding of the context-specific determinants of infant feeding practices is vital for the development of more effective BF promotion programs and informing local policies [36]. This may be particularly true for the Eastern Mediterranean Region (EMR), a region where the prevalence of EBF for the first six months postnatally does not exceed 30% [9, 37], and where data on the determinants of infant feeding practices are scarce [10, 11, 23, 29, 33].

To move this agenda forward, the ‘Mother and Infant Nutritional Assessment’ (MINA) cohort, was launched in 2015, as the first mother and child cohort in the EMR. It consists of a three year follow-up study of pregnant women and their children, in two Arab countries of the EMR, Lebanon and Qatar [38, 39]. Despite having discrepant income and development indicators, the prevalence of EBF is low in both Lebanon (12.3% in 2012, based on a questionnaire administered in a face-to-face interview) and Qatar (26% in 2019, based on a questionnaire administered via phone) [40, 41]. Using data stemming from the MINA cohort, the objectives of this study are to 1) characterize BF practices among the MINA cohort participants during the first six months postnatally and identify their correlates, 2) describe prenatal BF knowledge, attitude, exposure, and intention in the study sample, and 3) examine the association of prenatal BF knowledge, attitude, exposure, and intention with BF practices during the first six months postnatally.

## Methods

### Study design

Data for this study were derived from the MINA cohort conducted in Lebanon and Qatar. Details about the protocol and data collection of this cohort are described elsewhere [38]. Briefly, the MINA cohort is a three year longitudinal prospective study, where pregnant women are recruited during their first trimester. After delivery, women and their children are followed-up until the child is two years of age. Recruitment of subjects took place in various primary healthcare centers in Doha and Beirut. Over the course of the MINA cohort study, data collection was carried out in nine visits.

### Ethical considerations

The protocols used in the MINA cohort were reviewed and approved by two independent research ethics boards: the Institutional Review Board at the American University of Beirut (Protocol ID: NUT. FN. 12) and the Primary Health Care Corporation in Qatar (Protocol ID: PHCC / RC / 15 / 04 / 006). All MINA participants provided a written signed consent form. Subjects were reassured that their participation is completely voluntary, that they can withdraw at any time, and that their decision to continue or not in the study will not influence their provision of healthcare services.

### Study population

Subjects’ recruitment and data collection were performed in 2015 − 2018. To be eligible to participate, women had to be pregnant during their first trimester, pregnant with a singleton, of Lebanese or Qatari nationality, living in Lebanon or Qatar for more than five years, not planning to leave the current country of residence during the timeframe of the study, and not suffering from any chronic condition. In order to estimate the prevalence of exclusive BF, a total of 218 participants were needed for an effect size of 29%, a margin of error of 6% and a 95% confidence interval [42]. The 29% prevalence estimate was selected as an effect size as it reflected the average EBF for six months in the countries of the EMR [9].

### Study protocol and data collection

**Fig. 1:**
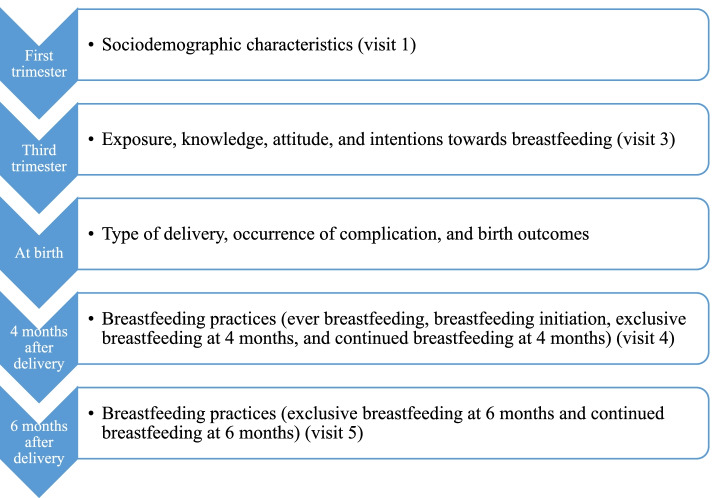
Data collection timeline for the MINA cohort

Through the MINA cohort, data collection was conducted during a one-to-one interview with the research personnel. All interviewers had received extensive training prior to the initiation of data collection in order to minimize interviewer errors. For the purpose of this study, data were extracted from visit 1 (first trimester), visit 3 (third trimester), the medical chart (at birth), visit 4 (four months after delivery), and visit 5 (six months after delivery) of the MINA cohort, as shown in Fig. 1. Below is a brief description of the data extracted from the MINA cohort and used in the study. Further details can be found at Naja et al. (2016) [38]:

- *Sociodemographic characteristics* (visit 1): age of the mother, number of children (excluding the current pregnancy), number of individuals living in the house, number of rooms in the house, education level, employment status, being related to husband, education level and employment status of the husband, and income. Crowding Index (CI), calculated as a ratio of the number of individuals living in the house over the number of the rooms in the house, was used as a proxy of socioeconomic status (SES) [43].

- *Delivery and infant characteristics* (medical charts)*:* type of delivery, occurrence of complications during delivery, gestational age classification of the offspring, and birth weight of the offspring (in grams); classified as either low birth weight (< 2500 g), normal weight (2500 − 4000 g) or macrosomic (> 4000 g) [44].

- *Exposure, knowledge, attitude, and intentions regarding* BF (visit 3): Information regarding exposure, knowledge, attitude, and intentions regarding BF were collected at visit 3. Prior exposure to BF was examined using the three questions proposed by Kavangh et al.: Ever been breastfed, knowing someone who has breastfed, and whether or not the participants have witnessed other women BF [31]. The answers to these questions were either yes (1 point) or no (0 points), except for ever been breastfed where a third option was given (unsure, also given 0 points). Using the total BF exposure score, participants were classified into low BF exposure (0 − 1 score) and high BF exposure (2 − 3 scores) [45]. The validated Arabic Breastfeeding Knowledge Questionnaire (BFK-A) was used to investigate the knowledge of participants with regards to BF [46]. Participants who gave the ‘correct’ or ‘wrong’ answers to any of the 20 questions were given a score of 1 or 0, respectively. The total BF knowledge score was categorized into: less than 9 (poor BF knowledge); 9 − 11 (fair knowledge); 12 − 13 (good knowledge) and higher than 14 (very good knowledge) [46]. The validated Arabic version of the IIFAS was used to explore the participants’ attitudes towards BF. The IIFAS consists of 17 items with a five-point Likert scale that ranges from 1 (strongly disagree) to 5 (strongly agree) [47, 48]. The total BF attitude score ranged from 17 to 85 and was classified as a strong positive attitude toward formula feeding (a score of 17 − 52), positive attitude toward formula feeding (a score of 53 − 59), neutral attitude (a score of 60 − 75), positive attitude toward BF (a score of 76 − 82), and strong positive attitude toward BF (a score of 83 − 85) [48]. BF intentions were examined using the validated Arabic Infant Feeding Intention (IFI) Scale. The IFI scale includes five infant feeding statements with a five-point Likert scale ranging from 0 (very much disagree) to 4 (very much agree) [49, 50]. The total score ranged from 0 to 16, and was classified into weak (0 to 7.5), fair (8 to 11.5), strong (12 to 15.5), and very strong (greater than 16) [50].

- *Infant feeding practices at four months after delivery* (visit 4): Data for the infant feeding practices were obtained during a one-to-one interview at the participant’s home and included information on ever BF, early initiation of BF, as well as current feeding practices [4]. The mother was asked if she is still breastfeeding her child and if yes, if her child is exclusively breastfed. BF was considered exclusive (EBF) when the mother reported that she has been exclusively feeding her infant with breast milk since birth, with no additional water, fluids, formula milk or foods [51]. The mother was also asked if she had practiced early initiation of breastfeeding, which was defined as putting the infant to the breast within one hour of birth [52]. *Infant feeding practices at six months after delivery* (visit 5): Information related to BF and EBF were obtained during the fifth visit, using similar protocol to that of the fourth visit.

### Statistical analysis

Frequencies and proportions as well as means ± standard deviation (SD) were used to described categorical and continuous variables, respectively. The feeding practices considered in this study were ever BF, BF initiation within the first hour after birth, BF at four months, EBF at four months, BF at six months, and EBF at six months. The answers of participants on each of the scales for exposure, knowledge, attitude, and intentions regarding BF were presented as frequencies and proportions. The total scores for knowledge, attitude, and intentions were computed and presented as means and SD as well as in categories as described earlier in this section. Simple and multiple logistic regressions were used to examine the determinants (sociodemographic and delivery characteristics) of feeding practices. The associations among exposure, knowledge, attitude, and intentions related to BF with feeding practices were also examined using simple and multiple logistic regression analyses. In all regression analyses the outcome variables were the feeding practices considered in this study. More specifically, the outcomes were ever BF (yes / no), BF initiation, EBF for four months, BF for four months and EBF for six months. For all analyses, predictor variables with a p-value of 0.2 in the simple regression were entered in the multiple regression models. All statistical analyses were carried out using Statistical Package for Social Sciences (SPSS) software 25 (SPSS Inc., Chicago, IL). P-values less than 0.05 were considered statistically significant.

### Results

**Fig. 2:**
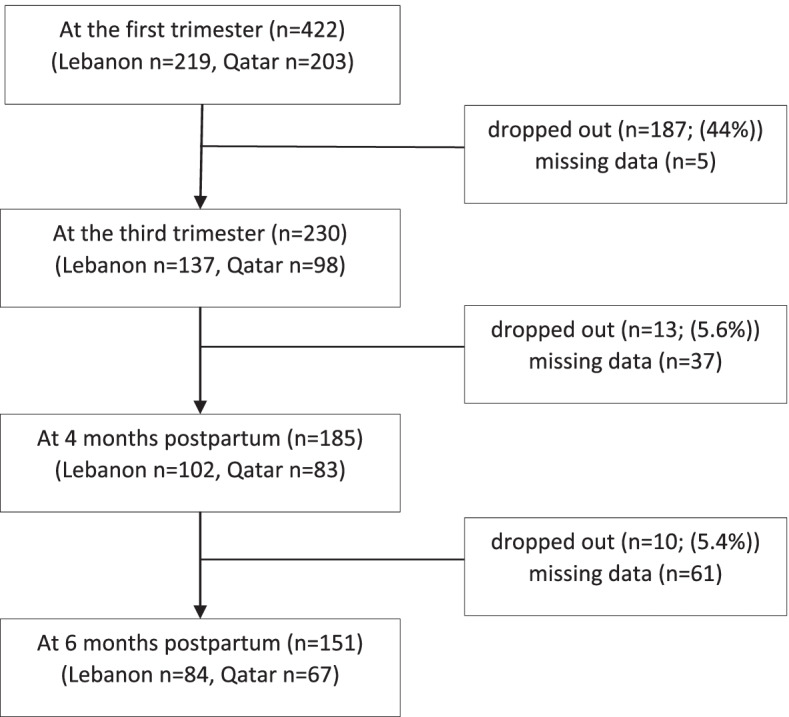
Flow chart describing the numbers of participants at each of the MINA cohort visits

A flow chart describing the numbers of participants at each of the visits is presented in Fig. 2. The largest dropout rate was observed after the first visit, with lower dropout rates being noted after the visits at four and six months postpartum (Fig. 2).

Table 1 displays the sociodemographic characteristics of participants who completed the third visit (*n* = 230). The study population consisted of 135 Lebanese and 95 Qatari pregnant women. Almost 38% of participants were 30 years of age or older, and 61% had one or more children (aside from the current pregnancy) (Table 1).

**Table 1:** Sociodemographic characteristics and delivery outcomes among the MINA Cohort participants (*n* = 230)

Sociodemographic characteristics	*n* (%)
**Country**	
Lebanon	135 (58.7)
Qatar	95 (41.3)
**Age**	
< 25 years	57 (25.8)
25 − 29.9 years	81 (36.7)
≥ 3 0 years	83 (37.6)
**Number of children** (excluding this pregnancy)	
None	90 (19.1)
1 or more	140 (60.9)
**Crowding index**	
< 1	108 (50.9)
≥ 1	104 (49.1)
**Education**	
Up to high school^a^	66 (30.4)
University or higher	151 (69.6)
**Health-related degree**	
No	113 (72)
Yes	44 (28)
**Employment status**	
Housewife	110 (50.5)
Employed (or student)	108 (49.5)
**Related to husband**	
Yes	32 (14.8)
No	184 (85.2)
**Husband’s education**	
Up to high school^a^	68 (31.6)
University or higher	147 (68.4)
**Income**	
Low, < 1000 US$	22 (16.2)
Middle, 1000 − 2000 US$	35 (25.7)
High, > 2000 US$	79 (58.1)
**Delivery outcomes**	
**Type of delivery**	
Cesarean section	57 (28.36)
Normal / vaginal	144 (71.6)
**Occurrence of any complications during delivery**	
No	113 (59.5)
Yes	77 (40.5)
**Gestational age classification**	
Full term	183 (92)
Preterm	16 (8)
**Birth weight classification**	
Low birth weight / macrosomia (< 2500 g / > 4000 g)	14 (7.03)
Normal birth weight (2500 − 4000 g)	185 (92.96)

Variables are presented as frequency and percentage [*n* (%)]

aIncluding technical diploma

**Fig. 3:**
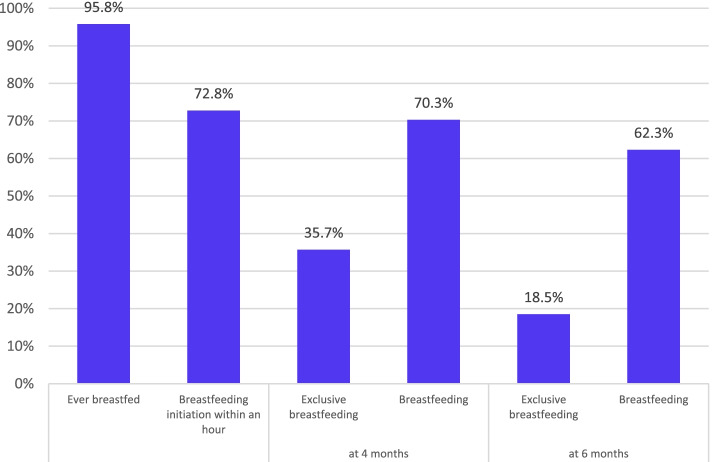
Feeding practices among the MINA cohort participants

Feeding practices in the study sample are presented in Fig. 3. The majority of participants indicated ever BF (95.8%), and 72.8% reported BF within the first hour after giving birth. Rates of BF were estimated at 70.3% at four months and 62.3% at six months, while those of EBF were estimated at 35.7% and 18.5% at four and six months, respectively (Fig. 3).

**Fig. 4:**
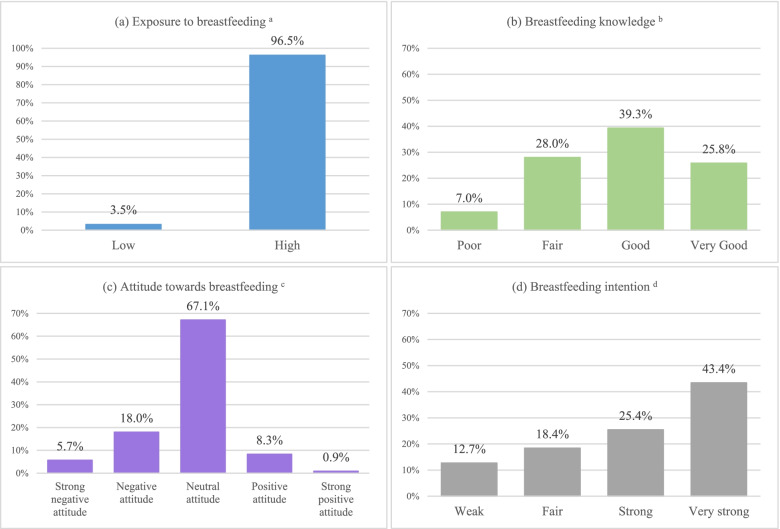
Distribution of breastfeeding (**a**) exposure, (**b**) knowledge, (**c**) attitude, and (**d**) intention among the MINA participants during the third trimester (*n* = 230) ^a, b, c, d^.aA score of 0 or 1 indicates low exposure to breastfeeding, and a score of 2 or 3 indicates high exposure (Hamade et al., 2014). bA score less than 9 indicates poor breastfeeding knowledge, 9 to 11 indicates fair knowledge, 12 to 13 indicates good knowledge, and greater than 14 indicates very good knowledge (Tamim et al., 2016). cA score of 17 − 52 indicates strong negative attitude toward breastfeeding, 53 − 59 indicates negative attitude toward breastfeeding, 60–75 indicates neutral attitude, 76 − 82 positive attitude toward breastfeeding, and 83 − 85 strong positive attitude toward breastfeeding. (Charafeddine et al., 2016). dA score of 0 − 7.5 indicates weak breastfeeding intention, 8 − 11.5 indicates fair intentions, 12 − 15.5 indicates strong intentions, and greater than 16 very strong intentions (Yehya et al., 2017)

The exposure to BF among participants is presented in Table 2 and Fig. 4a. High exposure to BF was observed among 96.5% of participants, with the lowest exposure reported for the question about ever been breastfed (86.1%). Table 3 details the answers of study participants to the BFK-A. The lowest proportions of correct answers were noted for the following two questions: ‘After a baby loses weight following birth, he / she will probably gain it back faster if’ (correct option: He / she is bottle-fed) and ‘Because babies may get a bad reaction to certain foods, BF mothers should never eat’ (correct option: none of the options is correct. Other important knowledge gaps were related to the best way to identify if the baby is getting enough milk; BF and its impact on the mother’s lifestyle; and the impact of BF on breast shape. Interestingly only half of the participating women provided a correct answer related to the ability of women to make enough milk to feed their baby and related to breast milk making up a complete diet for a baby. Less than half of the women identified factors that could lead to sore nipples. On the other hand, most of the mothers acknowledged that breast milk is the best food for the newborn, and that they should try to breastfeed even if they are planning to go back to work or school (Table 3). Overall, 25.8% of participants had very good knowledge while 7% had poor knowledge of BF (Fig. 4b).

**Table 2:** Exposure to breastfeeding during the third trimester among the MINA Cohort participants (n = 230)

	**MINA cohort participants**
	***n* (%)**
Ever been breastfed	198 (86.1)
Knows someone who has breastfed	221 (96.1)
Ever witnessed a woman breastfeeding	218 (94.8)

**Table 3:** Breastfeeding Knowledge among women of the MINA cohort (*n* = 230)

**The Infant Feeding Knowledge Test**^**a**^	**MINA Cohort Participants**
	***n* (%)**
Breastfeeding cuts down on the mother's bleeding after delivery (**True** False)	158 (68.7)
Breast milk makes up a complete diet for a baby (**True **False)	118 (51.3)
If your breasts are small, you might not have enough milk to feed the baby (True **False**)	192 (83.5)
When a mother is sick with the flu or a bad cold, she can usually continue to breastfeed her baby (**True** False)	155 (67.4)
Babies who are breastfed tend to get fewer allergies than babies who get formula (**True** False)	202 (87.8)
The pill is the best way to keep from getting pregnant while you are breastfeeding (True **False**)	156 (67.8)
You shouldn’t try to breastfeed if you are planning to go back to work or school since you won’t be able to be with your baby for feedings (True **False**)	204 (88.7)
The more often you breastfeed, the more milk you will have for your baby (**True** False)	209 (90.9)
Babies who are breastfed tend to get fewer infections than babies who get formula (**True** False)	209 (90.9)
Many women are not able to make enough milk to feed their baby (True **False**)	113 (49.1)
The best food for a newborn baby is: (**a. Breast milk** / b. Formula / c. Breast milk and water)	222 (96.5)
Because babies may get a bad reaction to certain foods, breastfeeding mothers should never eat: (a. Pizza or other spicy foods / b. Coffee, tea, or other drinks with caffeine / c. All of the above / **d. None of these are correct**)	44 (19.1)
After a baby loses weight following birth, he/she will probably gain it back faster if: (a. He/she is breastfed / **b. He/she is bottle-fed **/ c. Neither is correct)	42 (18.3)
You shouldn’t try to breastfeed if you: ^b^ (a. Have twins / b. Have a C-section / **c. Drink a lot of alcoholic beverages**)	131 (97)
Breastfeeding mothers’ nipples get sore if: (**a. The baby's feeding position is not right** / b. The mother has light-colored skin / c. This is the first baby she has breastfed)	96 (41.9)
When you breastfeed, the best way to tell if the baby is getting enough milk is by: (a. He / she does not suck on his / her fist after he / she is done nursing / b. He / she does not cry / **c. He / she has 6 or more wet diapers in 24 hours**)	63 (27.4)
When you breastfeed: (**a. You may get your figure back easier **/ b. You nearly always gain weight / c. You may feel weak when you feed your baby)	162 (70.4)
If you breastfeed: (a. No one else can help her with the baby since you have to feed him/her / b. More of your time will be taken up by the baby than if you bottle-feed / c. It will be very difficult to feed the baby in public places / **d. None of the above are correct**)	65 (28.3)
Breastfeeding will probably make: (a. Your breasts sag / b. Your breasts larger after you stop breastfeeding your baby / **c. No difference in the size or shape of your breasts**)	77 (33.5)
Breastfed babies need: (**a. Only breast milk for the first 4 to 6 months **/ b. A bottle of formula every day or so / c. Extra water on a daily basis)	186 (80.9)
***Infant Feeding Knowledge Test total score***^**c**^	***12.44 ± 2.26***

Categorical variables are presented as frequency and percentage; continuous variables are presented as mean ± SD

aThe Infant Feeding Knowledge Test consists of 10 multiple-choice questions and 10 true / false questions. The correct answers are presented in bold

bOnly asked of Lebanese participants

cThe Infant Feeding Knowledge Test total scores can range from 0 to 20, with a higher score indicating greater knowledge of breastfeeding

*SD* standard deviation

The attitudes and intentions to BF of study participants, as examined by the IIFAS and the IFI Scale are presented in Table 4 and Figs. 4c and 4d. For the attitude, the statements with the lowest means ± SD were ‘A mother who occasionally drinks alcohol should not breastfeed her baby’ and ‘The nutritional benefits of breast milk last only until the baby is weaned from breast milk’. In addition, more than half of the participants disagreed that formula feeding is the better choice for women who plan to work (52.8%) and that women should not breastfeed in public places (53.7%). The overall attitude score showed a sizeable proportion of participants (67.1%) reporting a neutral attitude, with only 1% displaying a strong positive attitude to BF and 5.7% having strong negative attitudes to BF (Fig. 4c). For the intention to breastfeed, 43.4% of women had very strong intentions to breastfeed, 25.4% had strong intentions, 18.4% fair intentions and 12.7% weak intentions (Fig. 4d).

**Table 4:** Breastfeeding attitude and intentions during the third trimester among the MINA Cohort participants (n = 230)

	**Score** **(Mean ± SD)**	**Agree/strongly agree**		**Neutral** **/ unsure**		**Disagree / strongly disagree**
		***n* (%)**	***n* (%)**		***n* (%)**	
The Iowa Infant Feeding Attitude Scale^a^						
The nutritional benefits of breast milk last only until the baby is weaned from breast milk^b^	3.29 ± 1.45	88 (38.4)	21 (9.2)		120 (52.4)	
Formula-feeding is more convenient than breastfeeding^b^	3.92 ± 1.3	43 (18.8)	11 (4.8)		175 (76.4)	
Breastfeeding increases mother / infant bonding	4.53 ± 0.86	212 (92.6)	7 (3.1)		10 (4.4)	
Breast milk is lacking in iron^b^	3.66 ± 1.12	36 (15.7)	60 (26.2)		133 (58.1)	
Formula fed babies are more likely to be overfed than are breastfed babies	3.43 ± 1.23	128 (55.9)	43 (18.8)		58 (25.3)	
Formula feeding is the better choice if the mother plans to work outside the home^b^	3.27 ± 1.33	80 (34.9)	28 (12.2)		121 (52.8)	
Mothers who formula feed miss one of the great joys of motherhood	3.83 ± 1.21	167 (72.9)	21 (9.2)		41 (17.9)	
Women should not breastfeed in public places such as in restaurants^b^	3.3 ± 1.3	72 (31.4)	34 (14.8)		123 (53.7)	
Babies fed breast milk are healthier than babies who are fed formula	4.15 ± 1.03	183 (80.3)	24 (10.5)		21 (9.2)	
Breastfed babies are more likely to be overfed than formula fed babies^b^	3.7 ± 1.17	41 (17.9)	40 (17.5)		148 (64.6)	
Fathers feel left out if a mother breastfeeds^b^	3.79 ± 1.13	35 (15.3)	36 (15.7)		158 (69)	
Breast milk is the ideal food for babies	4.69 ± 0.59	219 (95.6)	9 (3.9)		1 (0.4)	
Breast milk is more easily digested than formula	4.34 ± 0.91	197 (86)	23 (10)		9 (3.9)	
Formula is as healthy for an infant as breast milk^b^	4.04 ± 1.07	30 (13.1)	21 (9.2)		178 (77.7)	
Breastfeeding is more convenient than formula feeding	3.58 ± 1.37	142 (62)	22 (9.6)		65 (28.4)	
Breast milk is less expensive than formula	4.6 ± 0.68	214 (93.4)	11 (4.8)		4 (1.7)	
A mother who occasionally drinks alcohol should not breastfeed her baby^b^^, c^	2.09 ± 1.29	98 (73.1)	12 (9)		24 (17.9)	
*Iowa Infant Feeding Attitude Scale total score*	65.02 ± 7.74					
**The Infant Feeding Intentions Scale **^**d**^						
I am planning to only formula feed my baby^b^	3.63 ± 0.98	13 (5.7)	14 (6.1)		201 (88.2)	
I am planning to at least give breastfeeding a try	3.73 ± 0.89	215 (93.9)	2 (0.9)		12 (5.2)	
When my baby is 1 month old, I will be breastfeeding without using any formula or other milk	3.38 ± 1.23	183 (79.9)	19 (8.3)		27 (11.8)	
When my baby is 3 months old, I will be breastfeeding without using any formula or other milk	2.97 ± 1.43	150 (65.5)	35 (15.3)		44 (19.2)	
When my baby is 6 months old, I will be breastfeeding without using any formula or other milk	2.68 ± 1.49	125 (54.6)	53 (23.1)		51 (22.3)	
*Infant Feeding Intentions Scale total score*	12.70 ± 4.0					

^a^Attitude scale includes statements of a 5-point Likert-type scale ranging from 1 (strongly disagree) to 5 (strongly agree). These scores were grouped into the three categories: disagree (scores1 and 2), neutral and agree (scores 4 and 5). The Iowa Infant Feeding Attitude Scale total scores can range from 17 to 85, with higher scores representing a more positive attitude toward breastfeeding

^b^Question reverse-scored

^c^Only asked of Lebanese participants

^**d**^Intention scale includes statements of a 5-point Likert-type scale ranging from 0 (very much disagree) to 4 (very much agree). These scores were grouped into the following three categories: disagree (scores 0 and 1), unsure (score 2), and agree (scores 3 and 4). The Infant Feeding Intentions Scale total scores range from 0 to 16

*SD* standard deviation

The age-adjusted associations of sociodemographic and birth characteristics with BF practices, as derived from logistic regression, are presented in Table 5. After adjustment, using multiple regression, the following variables retained significance in predicting BF practices: Belonging to the Qatari arm of the cohort women were more likely to initiate BF as compared to their Lebanese counterparts (OR 3.47: 95% CI 1.07, 11.21); mothers having one or more children were more likely to continue BF until the fourth and sixth months (OR 2.82: 95% CI 1.19, 6.67 and OR 3.37; 95% CI 1.24, 9.21, respectively), while mothers having a crowding index of one or greater were less likely to do so (OR 0.31; 95% CI 0.13, 0.71 and OR 0.24; 95% CI: 0.09, 0.65, respectively); and employment was associated with lower odds of BF at four months (OR 0.43; 95% CI 0.2, 0.93). Women who experienced no complications during delivery were more likely to exclusively breastfeed at six months as compared to mother who had complications (OR 2.64; 95% CI 1.09, 6.44) (Data shown in Additional file 1).

**Table 5:** Age-adjusted associations of socio-demographic characteristics and birth outcomes (explanatory variables) and feeding practices (outcome variables)

	**Ever BF**		**Breastfeeding initiation**		**EBF for 4 months**		**Breastfeeding for 4 months**		**EBF for 6 months**		**Breastfeeding for 6 months**	
	**OR**	**CI 95%**	**OR**	**CI 95%**	**OR**	**CI 95%**	**OR**	**CI 95%**	**OR**	**CI 95%**	**OR**	**CI 95%**
**Sociodemographic characteristics**												
**Age** (ref: < 25)												
25 − 29.9	1.45	(0.28, 7.54)	1.42	(0.59, 3.42)	**0.43**	**(0.19**, **0.95)**	0.47	(0.2, 1.11)	0.91	(0.27, 3.13)	0.49	(0.2, 1.18)
≥ 30	2.28	(0.37, 14.23)	1	(0.44, 2.32)	0.72	(0.34, 1.56)	0.78	(0.32, 1.89)	2.59	(0.85, 7.93)	0.8	(0.32, 1.98)
**Country** (ref: Lebanon)												
Qatar	0.77	(0.18, 3.19)	**6.25**	**(2.58, 15.14)**	0.62	(0.33, 1.17)	1.19	(0.61, 2.3)	1.29	(0.55, 3.03)	1.16	(0.58, 2.32)
**Number of children** (excluding this pregnancy) (ref: None)												
1 or more	1.47	(0.32, 6.76)	**2.84**	**(1.33, 6.08)**	**2.37**	**(1.13**, **4.97)**	1.97	(0.96, 4.04)	1.69	(0.63, 4.56)	1.67	(0.8, 3.49)
**Crowding index** (ref: < 1 person / room)												
≥ 1 person / room	0.13	(0.02, 1.12)	**4.13**	**(1.94, 8.83)**	0.64	(0.34, 1.22)	0.62	(0.32, 1.22)	1.19	(0.5, 2.82)	0.65	(0.33, 1.32)
**Education** (ref: up to high school a)												
University or higher	1.21	(0.26, 5.69)	0.9	(0.41, 1.97)	1.04	(0.51, 2.14)	1.1	(0.52, 2.34)	0.74	(0.29, 1.92)	0.94	(0.43, 2.02)
**Health-related degree** (ref: No)												
Yes	1.61	(0.3, 8.51)	**2.46**	**(1.09, 5.55)**	0.63	(0.29, 1.38)	0.7	(0.29, 1.7)	0.88	(0.29, 2.67)	0.9	(0.36, 2.24)
**Employment** (ref: Housewife)												
Employed / Student	0.43	(0.09, 1.98)	**0.4**	**(0.19, 0.83)**	0.62	(0.33, 1.19)	0.6	(0.3, 1.17)	1.22	(0.51, 2.9)	**0.48**	**(0.24**, **0.97)**
**Related to husband** (ref: Yes)												
No	0.66	(0.08, 5.58)	0.38	(0.13, 1.17)	0.96	(0.42, 2.22)	0.71	(0.28, 1.8)	0.68	(0.24, 1.96)	0.6	(0.23, 1.57)
**Husband's educational level** (ref: up to high school)												
University or higher	0.75	(0.15, 3.87)	0.83	(0.39, 1.75)	1.23	(0.62, 2.45)	1.56	(0.77, 3.15)	1.21	(0.47, 3.07)	1.33	(0.64, 2.78)
**Income** (ref: Low, < 1000 US$)												
Medium, 1000 − 2000 US$	1.53	(0.08, 27.87)	0.48	(0.1, 2.28)	1.03	(0.27, 3.96)	0.88	(0.21, 3.7)	1.34	(0.21, 8.47)	0.74	(0.17, 3.3)
High, > 2000 US$	0.6	(0.06, 6.36)	0.5	(0.11, 2.17)	0.78	(0.23, 2.7)	1.25	(0.33, 4.76)	1.07	(0.19, 6.03)	0.93	(0.23, 3.71)
**Birth outcomes**												
**Type of delivery** (ref: Cesarean section)												
Normal / Vaginal	3.82	(0.8, 18.26)	**2.91**	**(1.39**, **6.1)**	1.12	(0.55, 2.3)	1.83	(0.89, 3.8)	2.37	(0.8, 6.96)	1.67	(0.78, 3.56)
**Occurrence of any complications during delivery** (ref: No)												
yes	0.49	(0.1, 2.56)	1.18	(0.57, 2.43)	0.8	(0.41, 1.6)	0.5	(0.24, 1.06)	0.61	(0.22, 1.67)	**0.41**	**(0.18**, **0.9)**
**Gestational age classification** (ref: Preterm)												
Full term	1.6	(0.18, 14.15)	2.13	(0.63, 7.15)	2.08	(0.54, 8.04)	1.03	(0.3, 3.58)	0.96	(0.19, 4.88)	0.93	(0.26, 3.41)
**Birth weight classification** (ref: Low birth weight / macrosomia)												
Normal birth weight (2500 − 4000 g)	2.24	(0.24, 20.98)	1.6	(0.49, 5.19)	0.68	(0.22, 2.16)	0.78	(0.2, 3.01)	0.39	(0.1, 1.58)	0.77	(0.19, 3.18)

Values in this table represent OR and their corresponding 95% CI, ORs with a bold font are statistically significant

aIncluding technical diploma

*BF* breastfeeding, *CI* confidence interval, *EBF* exclusive breastfeeding, *OR* odds ratio; ref: reference category

Multiple logistic regressions for the associations of exposure, knowledge, attitude, and intentions related to breastfeeding with feeding practices are presented in Table 6. A high exposure to breastfeeding was associated with greater odds of BF initiation. Knowledge about breastfeeding was not associated with any of the breastfeeding practices. Both positive attitude towards breastfeeding and strong intention to breastfeed were associated with EBF at four months, breastfeeding at four months, and breastfeeding at six months (Table 6).

**Table 6:** Multiple logistic regression analysis of the association between exposure to breastfeeding, breastfeeding knowledge, attitude towards breastfeeding, and intentions towards breastfeeding (explanatory variables) and feeding practices (outcome variables)

	**Ever BF**a		**Breastfeeding initiation**b		**EBF for 4 months**c		**Breastfeeding for 4 months**d		**EBF for 6 months**e		**Breastfeeding for 6 months**f	
**Exposure score** (ref: Low)												
High	4.25	(0.36, 50.6)	**10.1**	**(1.25, 80.65)**	1.28	(0.13, 12.7)	3.08	(0.5, 18.83)	0.49	(0.05, 5.18)	3.01	(0.45, 20.26)
**Infant Feeding Knowledge Test score** (ref: poor / fair)												
Good / Very good	2.74	(0.57, 13.05)	1.81	(0.73, 4.49)	1.14	(0.54, 2.41)	1.21	(0.58, 2.57)	1.00	(0.36, 2.76)	0.81	(0.32, 2.02)
**Iowa Infant Feeding Attitude Scale score** (ref: Positive attitude toward formula feeding)												
Neutral attitude / positive attitude towards breastfeeding	0.57	(0.06, 5.17)	1.33	(0.48, 3.7)	**2.51**	**(1.02, 6.16)**	**2.92**	**(1.29, 6.62)**	2.21	(0.55, 8.98)	**3.74**	**(1.24, 11.32)**
**Infant Feeding Intentions Scale score** (ref: weak / fair)												
Strong / very strong	1.59	(0.32, 7.89)	2.15	(0.84, 5.48)	**4.00**	**(1.67, 9.6)**	**5.00**	**(2.25, 11.1)**	2.56	(0.78, 8.36)	**8.29**	**(2.9, 23.68)**

Values in this table represent OR and their corresponding 95% CI, ORs with a bold font are statistically significant

aORs adjusted for age, country, crowding index, and delivery type

bORs adjusted for age, country, number of children, crowding index, health related degree, employment, and delivery type

cORs adjusted for age, country, number of children, crowding index and employment

dORs adjusted for age, number of children, crowding index, and employment

eORs adjusted for age, number of children, delivery type and birthweight

fORs adjusted for age, number of children, crowding index, employment, delivery type, and complications

*BF* breastfeeding, *CI* confidence interval, *EBF* exclusive breastfeeding, *OR* odds ratio; ref: reference category

## Discussion

This study is the first from the EMR to investigate maternal breastfeeding knowledge, attitude, and intention prenatally, and their association with actual feeding practices during the first six months postnatally. It showed that only 25% of women participating in the MINA cohort had very good knowledge, and 9.2% reported a positive / strong positive attitude towards breastfeeding, while the majority (96.5%) reported high previous exposure to breastfeeding. Even though the majority of participating women (70%) reported a strong intention to breastfeed, and actually initiated BF within the first hour after birth (72.8%), a sizable proportion could not meet the WHO recommendations in terms of EBF and breastfeeding duration. In fact, only 18.5% of participating women were able to exclusively breastfeed their baby for six months postpartum. The study showed that both a positive attitude towards breastfeeding and strong intention to breastfeed were independent predictors of EBF at four months, as well as breastfeeding at four and six months, while breastfeeding knowledge was not associated with any of the breastfeeding outcomes.

The observed high rate of early breastfeeding initiation in our cohort (72.8%) is comparable to previous estimates reported from Lebanon (77%) [50], while exceeding those reported from Qatar (57%) [53]. Despite these high initiation rates, the prevalence of EBF for six months was low, estimated at 18.5% in the study sample. This low prevalence confirms previous data described in Lebanon (10.1 − 26.6% EBF) [37, 40, 54, 55] and Qatar (18.9 − 26%) [37, 41], while showing that EBF rates in these two countries are lower than the average for the EMR as well as the global average (29.3% and 42%, respectively) [9, 37, 56]. The sharp decrease in the rates of EBF from 35.7% at four months to 18.5% at six months has been described by previous studies conducted in both Lebanon and Qatar [33, 57].

The study findings identified several sociodemographic attributes that were associated with infant feeding practices in the study sample. In agreement with previous studies conducted in Lebanon, Qatar [23, 33, 57], and elsewhere [34], maternal employment was associated with lower odds of breastfeeding. Similarly, women with a lower SES (as assessed by a crowding index ≥ 1) had lower odds of breastfeeding compared to those with higher SES, a finding that is in line with previous reports in the literature [58], highlighting the numerous social and environmental factors that contribute to the complex decision on infant feeding [10, 59]. The fact that participants from Qatar were more likely to initiate breastfeeding compared to their Lebanese counterparts may be a reflection of the higher SES in Qatar, or alternatively a reflection of the higher breastfeeding support within the hospital sites in Qatar compared to Lebanon [60]. In our study, mothers having one or more children were more likely to continue BF until the fourth and sixth month, which is consistent with earlier findings reported from Qatar [23], Lebanon [61], the United Arab Emirates (UAE) [62], as well as several other studies [63–65]. Higher parity may in fact be linked to better experience and enhanced maternal self-confidence, thus contributing to higher breastfeeding rates and longer breastfeeding duration [61].

Besides the demographic and socioeconomic factors that may modulate infant feeding decisions, it has been proposed that personal psychosocial factors and previous exposure to breastfeeding are important determinants of breastfeeding practices [27]. Several cross-sectional studies conducted among pregnant women have shown a direct association between breastfeeding exposure, knowledge, and attitude, with the intention to breastfeeding prenatally [20, 23, 29, 33]. However, few studies have investigated, longitudinally, the association between these maternal prenatal attributes and actual breastfeeding practices postnatally [22, 34]. Interestingly, the findings of our study showed that the proportions of women who had very good breastfeeding knowledge (25%) or positive / strong positive attitude towards breastfeeding (9.2%) were considerably lower than those practicing adequate breastfeeding practices such as early initiation of breastfeeding (72.8%) and breastfeeding at four and six months (70.3% and 62.3%).

In other instances in the literature, opposite findings were reported such as those described by Mogre et al. [66] and by Osibogun et al. [67], where high proportions of mothers displayed favorable breastfeeding knowledge and attitudes, but engaged in suboptimal breastfeeding practices. A direct association between breastfeeding practices and psychosocial factors is therefore not always observed, and the relationship between these constructs may be more complex than a simple lock-step relationship. Indeed, factors like breastfeeding protection and support as well as social normalization of breastfeeding may be more important than maternal breastfeeding knowledge [68]. In our study, antenatal breastfeeding knowledge was not associated with any of the investigated postnatal feeding practices, which lends further support to the complex relation between these constructs [69, 70]. In contrast, prior exposure to breastfeeding, which can be reflective of some form of social normalization of breastfeeding, was associated with a ten-fold increase in the odds of breastfeeding initiation among the cohort participants. Prior exposure to breastfeeding, such as the type of feeding women received from their own mothers, may in fact enhance the cultural acceptability of breastfeeding and contribute to more favorable attitudes towards this feeding modality [71, 72].

Our study findings showed that a positive attitude towards breastfeeding was associated with approximately a three-fold increase in EBF and breastfeeding at four months, and with a four-fold increase in the odds of breastfeeding at six months. In line with our findings, previous research has shown that improving maternal attitude and behavioral perceptions toward breastfeeding can significantly increase the likelihood of breastfeeding and improve its duration [73–76]. In the longitudinal US Infant feeding Practices Study II where breastfeeding attitude was assessed based on the perceived importance of EBF for the first six months, women who strongly valued EBF had more than twice the odds of EBF for three months and for six months compared to those with negative perceived value towards EBF [34]. A worrisome observation in our study is the fact that close to 70% of participating women displayed a neutral attitude towards breastfeeding, with only 1% displaying a strong positive attitude.

Recognizing that maternal attitude towards breastfeeding may be modifiable, these findings highlight the need for BF intervention programs that place a strong focus on prenatal breastfeeding attitudes and the value that mothers consign to breastfeeding [34]. In this context, our study identified specific items within the IIFAS that were particularly associated with a negative attitude, and that can be the target of future interventions. These essentially pertained to breastfeeding in public and the convenience of breastfeeding for a working mother, with more than half of participants stating that women should not breastfeed in public, and that formula feeding is more convenient than breastfeeding for working mothers. Interestingly, the negative attitude towards breastfeeding in public and towards the suitability of BF for working mothers were reported by a previous study conducted among undergraduate female students in Lebanon, showing how deep these negative perceptions are engraved within the local culture [45]. These negative attitudes may result from the dominating societal disapproval and the stigmatization of breastfeeding in public places, rendering it taboo in the Arab culture [45]. The negative attitude toward breastfeeding in working mothers, is worrisome, given that women are increasingly part of the labor force in both Lebanon and Qatar [33, 77].

In line with previous research showing that the intention to breastfeed is a well-established determinant of BF behavior, and particularly EBF [34, 78, 79], our study showed that a strong intention to breastfeed was associated with a three-fold increase in the odds of EBF and breastfeeding at four months, and with a four-fold increase in the odds of breastfeeding at six months. Hamade et al. [36] have also previously highlighted the intention to breastfeed as one of the significant predictors of EBF among Lebanese mothers. In a study conducted in the US, DiGirolamo et al. investigated the effects of prenatal intention on breastfeeding initiation and duration and showed that prenatal intention was a significant predictor of positive BF practices postnatally [79]. However, the study by DiGirolamo et al. also showed that, in addition to prenatal intention, the initial breastfeeding experiences of the mother were significantly associated with breastfeeding outcomes, and particularly with early termination.

This may explain the intention vs behavior gap that was reported by several previous investigations [22, 34], and was also a main observation in our study. In fact, although close to 70% of women participating in the MINA cohort had expressed a strong or very strong intention to breastfeed their baby, only 18.5% had exclusively breastfed for six months and 35.7% for four months. These findings suggest that women’s initial intention, assessed during pregnancy, can potentially change throughout the six months period postnatally. Mothers may in fact be challenged postnatally, by certain environmental barriers to breastfeeding such as the lack of support at home, workplace, or hospitals [22, 34] or by emotional and psychological barriers [22, 79]. According to Rothman’s (2000) [80], the factors that lead to behavioral intention or initiation differ from those leading to behavioral maintenance, the latter being influenced not only by intention but also by perceived satisfaction with the outcome [80]. For example, DiGirolamo et al. [79] showed that women with a relatively negative initial experience with BF, such as problems or complications during the first week or a reported lack of comfort, were less likely to continue breastfeeding by ten weeks postpartum. In a qualitative study, Ahishakiye et al. also reported that postnatal discomfort, personal confidence in the ability to breastfeed, and perceived breastmilk insufficiency were among the factors that modulated breastfeeding behavior postnatally [22].

The possibility that environmental or psychological barriers may emerge and affect breastfeeding behavior postnatally may also explain why, in our study, breastfeeding knowledge, which was previously shown to be associated with breastfeeding intention or positive breastfeeding outcomes [74–83] was not found to predict breastfeeding behavior among MINA participants. There is a need for future studies that provide an-in-depth appraisal of factors that could lead to improvements in BF outcomes postnatally and identify context-specific barriers and facilitators.

The strengths of this study include its prospective nature, thus minimizing recall bias that is often associated with cross-sectional studies. Furthermore, despite the MINA cohort being a multi-country cohort, the study protocols and data collection procedures were standardized across both study sites [38]. However, the results of this study ought to be considered in view of the following limitations. First, the small sample size in our cohort may have led to underpowered analyses. Second, psychosocial characteristics and feeding practices were assessed using questionnaires that were administered in an interview setting. As is the case with most questionnaire-based studies, the interview-based approach may result in social desirability bias [84]. In our study, all interviewers had received extensive training prior to the initiation of data collection in order to minimize judgmental verbal and nonverbal communication and consequently reduce the likelihood of social desirability bias.

## Conclusions

In conclusion, this study documented suboptimal knowledge and attitude towards breastfeeding in a sample of Middle Eastern women, coupled with low rates of EBF. While knowledge was not associated with breastfeeding practices, a high prior exposure, a positive breastfeeding attitude, and a strong intention to breastfeed prenatally were significant predictors of breastfeeding practices postnatally. Interestingly, although close to 70% of women had expressed a strong intention to breastfeed, only 18.5% had exclusively breastfed for six months, a finding that may reflect the challenges encountered postnatally by women, and which often include poor self-efficacy, and / or lack of support at home, workplace, or hospitals [85].

Taken together, the study findings highlight the need for developing specific interventions and policies aimed at protecting, supporting and normalizing breastfeeding as a social norm, and improving breastfeeding attitudes among women, while tailoring these interventions to the local context and culture. By abating societal taboos and promoting breastfeeding, such interventions may play a central role in addressing the prevalent negative attitudes such as the issue of BF in public and the suitability of breastfeeding for a working mother. The study results also highlight the need to better understand what influences prenatal breastfeeding intentions, given that it was shown to be an important predictor of a mother’s behavior after delivery [79]. It is through the investment in BF and enhanced infant nutrition that countries step towards the path of building their human capital, developing their economies, and shaping their future prosperity [86].

## Supplementary Information

**Additional file 1.** Multiple logistic regression analysis of the association between socio-demographic characteristics and birth outcomes (explanatory variables) and feeding practices (outcome variables). Values in this table represent OR and their corresponding 95%CI,ORs with a bold font are statistically significant. ^a^Includingtechnical diploma.*BF*breastfeeding, *CI* confidence interval, *EBF* exclusivebreastfeeding, *OR* odds ratio; ref: reference category

## Data Availability

The datasets used and / or analysed during the current study are available from the corresponding author on reasonable request.

## Abbreviations

BF: Breastfeeding
BFK-A: Breastfeeding Knowledge Questionnaire
CI: Confidence interval
CI: Crowding index
EBF: Exclusive breastfeeding
EMR: Eastern Mediterranean Region
IFI: Infant Feeding Intention
IIFAS: Iowa Infant Feeding Attitude Scale
MINA: Mother and Infant Nutritional Assessment
NCDs: Non-communicable diseases
SD: Standard deviation
SES: Socioeconomic status
UAE: United Arab Emirates
US: United States
WHO: World Health Organization
