# Supplementary material for: Prenatal breastfeeding knowledge, attitude and intention, and their associations with feeding practices during the first six months of life: a cohort study in Lebanon and Qatar
Source: Int Breastfeed J. 2022 Feb 24;17:15. doi: 10.1186/s13006-022-00456-x (PMC8867651; doi:10.1186/s13006-022-00456-x)
Supplement: Supplementary file 1 — Additional file 1. Multiple logistic regression analysis of the association between socio-demographic characteristics and birth outcomes (explanatory variables) and feeding practices (outcome variables). Values in this table represent OR and their corresponding 95%CI,ORs with a bold font are statistically significant. aIncludingtechnical diploma.BFbreastfeeding, CI confidence interval, EBF exclusivebreastfeeding, OR odds ratio; ref: reference category [file 13006_2022_456_MOESM1_ESM.docx]

|  | **Ever BF** | | **Breastfeeding initiation** | | **Excusive BF for 4 months** | | **Continuous feeding for 4 months** | | **Exclusive BF for 6 months** | | **Continuous feeding for 6 months** | |
| --- | --- | --- | --- | --- | --- | --- | --- | --- | --- | --- | --- | --- |
| **Sociodemographic** |  |  |  |  |  |  |  |  |  |  |  |  |
| **Age** (ref: <25) |  |  |  |  |  |  |  |  |  |  |  |  |
| 25-29.9 | 1.67 | (0.3, 9.44) | 1.36 | (0.46, 3.98) | 0.32 | (0.13, 0.82) | 0.4 | (0.15, 1.1) | 0.92 | (0.26, 3.28) | 0.39 | (0.13, 1.21) |
| ≥30 | 5.3 | (0.5, 56.21) | 0.57 | (0.18, 1.79) | 0.48 | (0.18, 1.26) | 0.52 | (0.18, 1.54) | 2.12 | (0.62, 7.25) | 0.5 | (0.14, 1.77) |
| **Country** (ref: Lebanon) |  |  |  |  |  |  |  |  |  |  |  |  |
| Qatar |  |  | **3.47** | **(1.07, 11.21)** | 0.59 | (0.24, 1.43) |  |  |  |  |  |  |
| **Number of children** (excluding this pregnancy) (ref: None) | | |  |  |  |  |  |  |  |  |  |  |
| 1 or more |  |  | 2.01 | (0.79, 5.16) | 3.4 | (1.47, 7.88) | **2.82** | **(1.19,** **6.67)** | 1.9 | (0.65, 5.51) | **3.37** | **(1.24, 9.21)** |
| **Crowding Index** (ref: <1 person/room) |  |  |  |  |  |  |  |  |  |  |  |  |
| ≥1 person/room | 0.12 | (0.01, 1.04) | 1.75 | (0.64, 4.8) | 0.47 | (0.19, 1.15) | **0.31** | **(0.13, 0.71)** |  |  | **0.24** | **(0.09, 0.65)** |
| **Education** (ref: up to high school ^a^) |  |  |  |  |  |  |  |  |  |  |  |  |
| University or higher |  |  |  |  |  |  |  |  |  |  |  |  |
| **Health-related degree** (ref: Yes) |  |  |  |  |  |  |  |  |  |  |  |  |
| Yes |  |  | 2.18 | (0.84, 5.69) |  |  |  |  |  |  |  |  |
| **Employment** (ref: Housewife) |  |  |  |  |  |  |  |  |  |  |  |  |
| Employed/Student |  |  | 1.28 | (0.49, 3.35) | 0.52 | (0.24, 1.1) | **0.43** | **(0.2, 0.93)** |  |  | 0.42 | (0.17, 1.07) |
| **Related to husband** (ref: Yes) |  |  |  |  |  |  |  |  |  |  |  |  |
| No |  |  |  |  |  |  |  |  |  |  |  |  |
| **Husband's educational** **level** (ref: up to high school) |  |  |  |  |  |  |  |  |  |  |  |  |
| University or higher |  |  |  |  |  |  |  |  |  |  |  |  |
| **Income** (ref: Low, <1000$) |  |  |  |  |  |  |  |  |  |  |  |  |
| Medium, 1000$-2000$ |  |  |  |  |  |  |  |  |  |  |  |  |
| High, >2000$ |  |  |  |  |  |  |  |  |  |  |  |  |
| **Birth Outcomes** |  |  |  |  |  |  |  |  |  |  |  |  |
| **Type of delivery** (ref: Caesarean section) |  |  |  |  |  |  |  |  |  |  |  |  |
| Normal/Vaginal | 4.77 | (0.95, 23.86) | 2.08 | (0.88, 4.9) |  |  |  |  | 2.00 | (0.67, 6.02) | 1.38 | (0.57, 3.35) |
| **Occurrence of any complications during delivery** (ref: Yes) | | |  |  |  |  |  |  |  |  |  |  |
| No |  |  |  |  |  |  |  |  |  |  | **2.64** | **(1.09, 6.44)** |
| **Gestational age classification** (ref: Preterm) |  |  |  |  |  |  |  |  |  |  |  |  |
| Full term |  |  |  |  |  |  |  |  |  |  |  |  |
| **Birth weight classification** (ref: Low birth weight/macrosomia) | | |  |  |  |  |  |  |  |  |  |  |
| Normal birth weight (2500-4000 g) |  |  |  |  |  |  |  |  | 0.45 | (0.11, 1.85) |  |  |
